# Low-Light Image Enhancement Using Adaptive Digital Pixel Binning

**DOI:** 10.3390/s150714917

**Published:** 2015-06-25

**Authors:** Yoonjong Yoo, Jaehyun Im, Joonki Paik

**Affiliations:** 1 Image Processing and Intelligent Systems Laboratory Graduate School of Advanced Imaging Science, Multimedia, and Film Chung-Ang University, Seoul 156-756, Korea; E-Mail: whitener@cau.ac.kr; 2 CIS Division, SK Hynix, Gyeonggi-do 463-844, Korea; E-Mail: jaehyun2.im@sk.com

**Keywords:** pixel binning, image enhancement, anti-saturation

## Abstract

This paper presents an image enhancement algorithm for low-light scenes in an environment with insufficient illumination. Simple amplification of intensity exhibits various undesired artifacts: noise amplification, intensity saturation, and loss of resolution. In order to enhance low-light images without undesired artifacts, a novel digital binning algorithm is proposed that considers brightness, context, noise level, and anti-saturation of a local region in the image. The proposed algorithm does not require any modification of the image sensor or additional frame-memory; it needs only two line-memories in the image signal processor (ISP). Since the proposed algorithm does not use an iterative computation, it can be easily embedded in an existing digital camera ISP pipeline containing a high-resolution image sensor.

## 1. Introduction

As the demand for mobile devices increases, the density of a CMOS image sensor is rapidly growing. However, if the size of each pixel in the high-density image sensor becomes smaller, low-sensitivity and noise amplification problems occur, especially in low-light images. To solve this problem, many image signal processors (ISPs) adopt digital image enhancement algorithms. Since a simple intensity amplification algorithm results in various undesired artifacts, sensor-based pixel binning [1,2,3,4] algorithms with noise reduction function in the image signal processor (ISP) [5,6,7] have been proposed in the literature. More specifically, a basic sensor-based pixel binning method groups multiple neighboring pixels into one to increase the sensitivity of the resulting pixel at the cost of reduced spatial resolution. In order to reduce the side effect of brightness amplification, various noise reduction algorithms were also proposed to separate noise from the original image before brightness amplification. However, a basic sensor-based pixel binning method cannot avoid loss of spatial resolution, and noise reduction is still an open problem in the imaging sensor technology. The proposed work extends the digital pixel binning algorithm in a context-adaptive manner to prevent undesired artifacts including noise amplification, intensity saturation, and loss of resolution.

This paper is organized as follows. A novel image degradation model for low-light image acquisition and the related background theory are presented in Section 2, and the proposed adaptive digital pixel binning algorithm is presented in Section 3. Experimental results are given in Section 4, and Section 5 concludes the paper.

## 2. Theoretical Background

**Figure 1:**
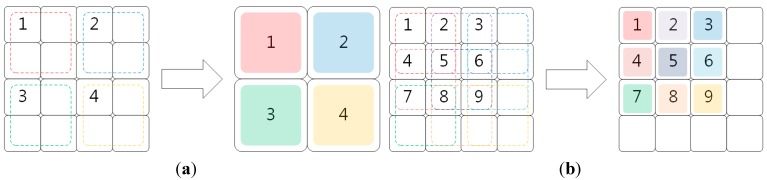
Two different pixel binning approaches to combine 2 × 2 pixels into one bin: (**a**) sensor-based pixel binning; and (**b**) digital pixel binning.

Sensor-based pixel binning is designed to increase the sensitivity of an image sensor by combining multiple photodiodes into one bin at the cost of decreasing the spatial resolution as shown in Figure 1a. On the other hand, digital pixel binning can accumulate multiple pixel values without losing the spatial resolution as shown in Figure 1b.

Since the sensor-based pixel binning method does not reuse combined pixels as shown in Figure 1a, it decreases the spatial resolution inversely proportional to the bin size. On the other hand, the digital pixel binning method reuses pixels used for adjacent bins as shown in Figure 1b. The result of digital pixel binning can be considered a low-pass filtered version of the input image whose intensities are multiplied by the bin size. For this reason, spatial resolution is preserved in the pixel binning process while the details are smoothed by low-pass filtering.

### 2.1. Image Degradation Model for Low-Light Image Acquisition 

**Figure 2:**
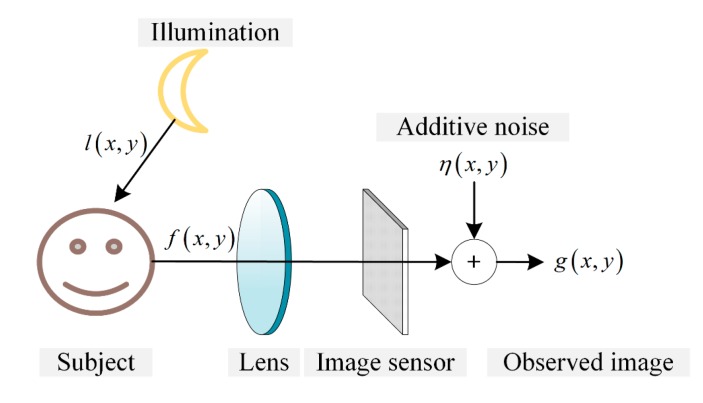
Image degradation model with an image sensor under low-light conditions.

The proposed image degradation model under low-light conditions is shown in Figure 2, where l(x,y) and f(x,y) represent the luminance of the low-light environment and the reflected luminance by the subject, respectively. 

The degraded image under low-light conditions can be expressed as:

(1) g(x,y)=f(x,y)+η(x,y)

Since g(x,y) is a low-light version, a simple intensity amplification by factor α yields:

(2) α·g(x,y)=α·f(x,y)+α·η(x,y)

Although αg(x,y) has an amplified intensity, it results in intensity saturation because of the limited bit range, and also amplifies the sensor noise.

### 2.2. Digital Pixel Binning

The digital pixel binning process is expressed as:

(3) $$\hat{g}(x,y)=\alpha \cdot h_{b}\left(x,y\right)*g\left(x,y\right)=\alpha \cdot h_{b}\left(x,y\right)*\left\{f\left(x,y\right)+\eta \left(x,y\right)\right\}$$

where $h_{b}\left(x,y\right)$ represents the binning kernel, and * represents the convolution operation. For example, the kernel of 2 × 2 pixels binning plays a role of average filtering as:

(4) $$h_{b}=\left[\begin{array}{cc} 1 & 1\\ 1 & 1 \end{array}\right]/\alpha$$

where α = 4. Given a specific value of α, the result of pixel binning in Equation (3) is rewritten as:

(5) $$\hat{g}\left(x,y\right)=\alpha \cdot h_{b}\left(x,y\right)*f\left(x,y\right)+\alpha \cdot \hat{\eta }\left(x,y\right)$$

where $\hat{\eta }\left(x\right)=h_{b}\left(x,y\right)*\eta \left(x,y\right)$. In $\hat{g}\left(x,y\right)$, the high frequency detail of the low-light degraded version, that is f(x,y), is suppressed by the average filtering, and then amplified by the ratio α. The distribution of random noise in $\hat{g}\left(x,y\right)$ is concentrated toward zero by the average filtering, and will not be affected by the following amplification by the ratio α since most samples has become close to zero.

**Figure 3:**
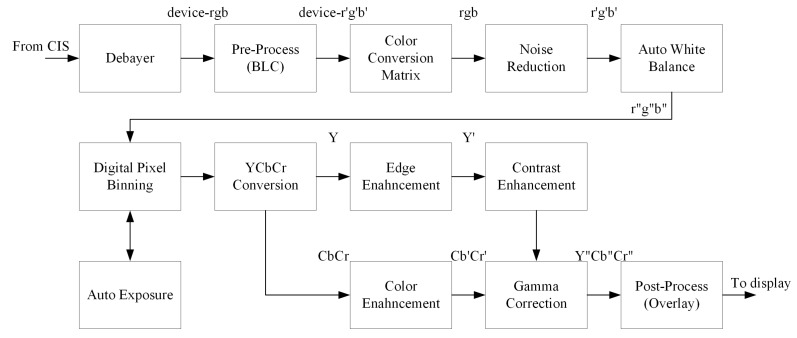
Block diagram of the ISP pipeline including the digital pixel binning function.

The digital pixel binning function in the conventional ISP for surveillance is shown as Figure 3.

Digital pixel binning amplifies the intensity value before auto exposure (AE). The rest of the ISP functions, including noise reduction and image enhancement, can be used together with digital binning.

The digital pixel binning algorithm can be easily implemented using additional line memories. However, simple digital pixel binning algorithm results in image degradation as given in Equation (5). The proposed binning algorithm has been devised to avoid image degradation and additional hardware. 

## 3. Adaptive Digital Pixel Binning

**Figure 4:**
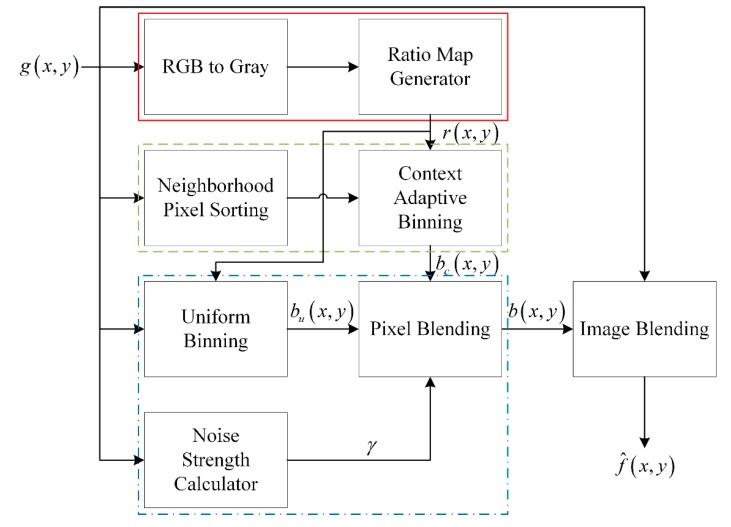
The proposed adaptive digital pixel binning algorithm.

Although digital pixel binning can produce a brighter image without noise amplification in low-light conditions, the resolution of the resulting image decreases because of the low-pass filtering nature of the binning kernel. Furthermore, the intensity of the bright region is saturated when the pixel binning uses a fixed ratio. To solve these problems, the proposed algorithm performs the binning process in an adaptive manner according to the brightness, context, noise, and anti-saturation. Figure 4 shows the block diagram of the proposed adaptive digital pixel binning algorithm.

### 3.1. Brightness Adaptive Binning Ratio

The ordinary digital pixel binning method amplifies the brightness of entire image using a fixed amplification ratio. For this reason, the dark region of image is enhanced to be brighter, while the bright region becomes saturated. To solve the saturation problem of conventional digital binning algorithms, the proposed algorithm changes the amplification ratio according to the brightness of the neighboring region in a spatially adaptive manner. The optimal amplification ratio at each pixel using the converted gray channel and the 3 × 3 average filter to suppress noise can be determined as follows:

(6) $$t\left(x,y\right)=\frac{h_{3\times 3}*\tilde{g}\left(x,y\right)}{\text{max}\left\{h_{3\times 3}*\tilde{g}\left(x,y\right)\right\}}$$

where $h_{3\times 3}$ represents the 3 × 3 average filter, $\tilde{g}\left(x,y\right)$ the converted gray cannel image, and max{·} the operation that selects the maximum value in the argument. As a result, t(x,y) has the value in [0, 1], and the order of relative brightness is preserved. The optimal binning ratio is defined using t(x,y) as:

(7) $$r\left(x,y\right)=1+\left(1-t\left(x,y\right)\right)\left(R_{b}-1\right)$$

where $R_{b}$ represents the maximum binning ratio. r(x,y) takes the value 1 or higher. If t(x,y) becomes close to 0, r(x,y) approaches to its maximum value $R_{b}$. For example, if $R_{b}$ is set to 4, the proposed algorithm uses the maximum of four pixels. 

### 3.2. Context-Adaptive Binning

The pixel averaging process in the binning algorithm decreases the resolution of the resulting image. To minimize such degradation, the proposed algorithm uses weighted summation based on the relationship between the pixel and its neighbor. At the pixel position (x,y), a p×p window generates the sorted difference vector $d_{x,y}\in R^{p^{2}}$ as:

(8) $$d_{x,y}=\text{sort}\left\{g\left(x,y\right)-g\left(x+i,y+j\right)\right\},\;\text{for}\;i,j=-\left\lfloor p/2\right\rfloor ,\ldots ,\left\lfloor p/2\right\rfloor$$

where ⌊p/2⌋ represents the integer part of p/2, and sort{·} represents the vector whose elements are rearranged in the ascending order of the absolute value such that $\left|d_{x,y}(1)\right|\leq \left|d_{x,y}(2)\right|\leq \cdots \leq \left|d_{x,y}(p^{2})\right|$ Based on the definition in Equation (8), $d_{x,y}(1)$ is always equal to zero. Each element can have either a positive or negative value, whereas the absolute values are used for only sorting. The sorted vector of the p×p window based on the absolute difference from the center pixel is defined as:

(9) $$s_{x,y}=g(x,y)-d_{x,y}$$

The result of the context-adaptive binning is obtained from the weighted sum of similar pixels in the sorted vector as:

(10) $$b_{c}\left(x,y\right)=r_{s}^{T}s_{x,y}$$

where the weighting vector $r_{s}$ is defined using the optimal binning ratio given in Equation (7) as:

(11) $$r_{s}\left(q\right)=\{\begin{array}{ll} 1, & r\left(x,y\right)-\left(q-1\right)>1\\ r\left(x,y\right)-\left(q-1\right), & 0<r\left(x,y\right)-\left(q-1\right)\;\leq 1\\ 0, & \text{otherwise} \end{array}$$

The pixel order *q* represents the labeling of each sorted pixel in the p×p window. 

**Figure 5:**
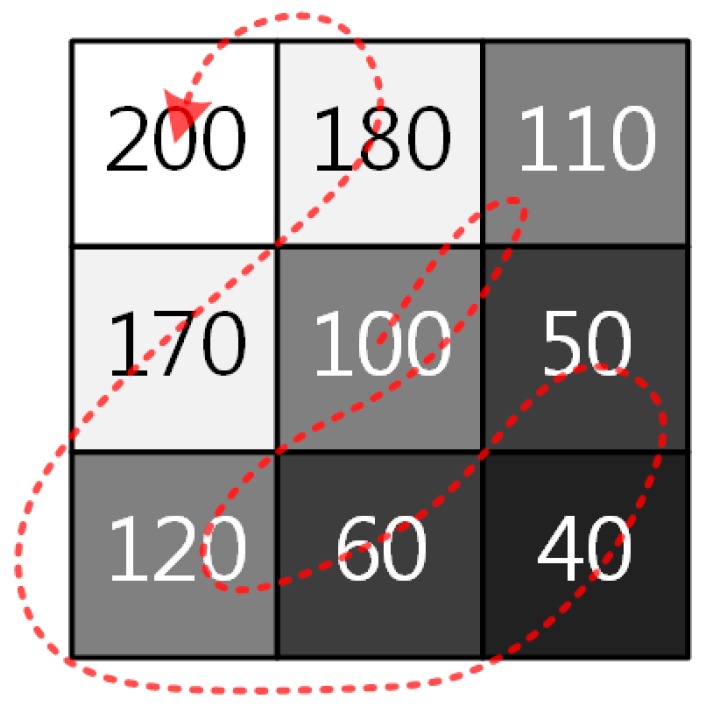
An example of a 3×3 window with g(x,y)=100 at the center.

For example, a 3×3 window is shown in Figure 5, where p=3 and g(x,y)=100. The corresponding $d_{x,y}$ and $s_{x,y}$ are respectively computed as:

(12) $$d_{x,y}={\left[\begin{array}{ccccccccc} 0 & -10 & -20 & 40 & 50 & 60 & -70 & -80 & -100 \end{array}\right]}^{T}$$

and

(13) $$s_{x,y}={\left[\begin{array}{ccccccccc} 100 & 110 & 120 & 60 & 50 & 40 & 170 & 180 & 200 \end{array}\right]}^{T}$$

If r(x,y)=3.4, and q=4, the sorted ratio values are determined as $r_{s}(1)=r_{s}(2)=r_{s}(3)=1.0$, and $r_{s}(4)=0.4$. The binning result is then finally given as:

(14) $$b_{c}\left(x,y\right)=(1.0\times 100)+(1.0\times 110)+(1.0\times 120)+(0.4\times 60)=354$$

The kernel of the adaptive binning is expressed in matrix form as:

(15) $$K_{c}=\left[\begin{array}{ccc} 0 & 0 & 1\\ 0 & 1 & 0\\ 1 & 0.4 & 0 \end{array}\right]$$

Since three pixel values 110, 100, 120 in the diagonal edge are similar as shown in Figure 5, only summation of the three pixels can preserve the edge without being mixed with uncorrelated pixels, and the fourth similar pixel value, that is 60, is combined with smaller weight than the first three.

The ordinary digital pixel binning sums up pixel values in the pre-defined binning kernel throughout the input image. For this reason, details of the image cannot be preserved. On the other hand, the proposed algorithm can flexibly change the binning kernel based on the correlation of neighboring pixels. As a result, it is possible to minimize the degradation of edge details in the input image. 

### 3.3. Noise-Adaptive Pixel Binning

Although the proposed context adaptive pixel binning can preserve the details in the image, it cannot suppress noise if the current pixel contains a noisy component. For this reason, uniform binning is effective in a noisy region as:

(16) $$b_{u}\left(x,y\right)=r_{u}^{T}s_{x,y}$$

where $r_{u}$ represents the uniform pixel binning coefficient vector whose elements are defined as:

(17) $$r_{u}\left(q\right)=\{\begin{array}{cc} 1, & \text{if}\;q=1\\ \frac{1}{p^{2}-1}\left\{r\left(x,y\right)-1\right\}, & \text{otherwise} \end{array}$$

The unity value of *q* implies the current pixel whose position coefficient is equal to 1. However, coefficients of other pixels are determined by the number of total pixels to reduce the noise effect. If r(x,y)=3.4, the uniform binning kernel is expressed in matrix form as:

(18) $$K_{u}=\left[\begin{array}{ccc} 0.3 & 0.3 & 0.3\\ 0.3 & 1 & 0.3\\ 0.3 & 0.3 & 0.3 \end{array}\right]$$

The low-pass filtering performance of this kernel is higher than ordinary four-pixel digital pixel binning because its kernel has greater support. The result of the proposed binning is computed as:

(19) $$b\left(x,y\right)=\left(1-\gamma \right)\cdot b_{c}\left(x,y\right)+\gamma \cdot b_{u}\left(x,y\right),\;\text{for}\;\gamma =\frac{1}{\lambda }\left|\bar{s}\left(q\right)-s\left(1\right)\right|$$

where $\bar{s}\left(q\right)$ represents the mean value of the local window, and λ a constant for the sensitivity of noise suppression. In this work, λ=1 was used for empirically best result. 

### 3.4. Image Blending for Anti-Saturation

Although the brightness-adaptive procedure adjusts the binning ratio, the binning algorithm itself simply amplifies the brightness and results in saturation in the processed image. In order to prevent saturation, the proposed algorithm combines the input low-light and the binning result given in Equation (19) using the image blending method.

The blending image is computed by combining the amplified image b(x,y) and the input g(x,y) as:

(20) $$\hat{f}\left(x,y\right)=\left(1-w\left(x,y\right)\right)\cdot b\left(x,y\right)+w\left(x,y\right)\cdot g\left(x,y\right)$$

where the blending weighted coefficient is defined as:

(21) $$w\left(x,y\right)=\frac{1}{\mu }\left\{\frac{b\left(x,y\right)}{R_{b}-1}+\frac{g\left(x,y\right)}{2}\right\}$$

In Equation (9), μ represents the maximum bit depth of the image for normalization. If the maximum binning ratio increases, w(x,y) becomes smaller because b(x,y) is divided by $\left(R_{b}-1\right)$. For this reason, the saturated value is substituted by the lower one. If w(x,y) increases, $\hat{f}\left(x,y\right)$ becomes closer to g(x,y), which is the input image in low-light environments. On the other hand, if w(x,y) decreases, the corresponding region becomes darker. In this case, $\hat{f}\left(x,y\right)$ takes the data from the amplified image b(x,y). As a result, $\hat{f}\left(x,y\right)$ is the image of suppressed noise and saturation.

## 4. Experimental Results

Three test images were acquired using two different types of camera for the experiment. A digital camera with a 20 megapixel CMOS image sensor was used to acquire two groups of test images: (1) a resolution chart; and (2) a parking garage. A commercial surveillance camera with a full high-definition (1920 × 1080) CMOS image sensor was also used to capture the outdoor scene. In order to obtain images with different exposure values (EVs), multiple ISO values were used with a fixed aperture and shutter speed. For evaluation of the performance of noise suppression, entropy and variance values are computed in a flat region of images. In order to acquire input test images, auto white balance (AWB) and AE parameters are fixed, and edge enhancement and color suppression functions are bypassed. In order to evaluate the independent performance of digital pixel binning, noise reduction functions were turned off, and only gamma correction is applied as shown in Figure 3.

In order to evaluate the performance of brightness enhancement, the proposed algorithm is compared with ordinary digital pixel binning, Kim’s clipped histogram equalization [8], and Jiang’s image enhancement [9]. Each amplification ratio of image enhancement algorithms was set to preserve the same brightness as the reference value. However, the average brightness of output images is not exactly the same because of the different properties of each amplification algorithm.

**Figure 6:**
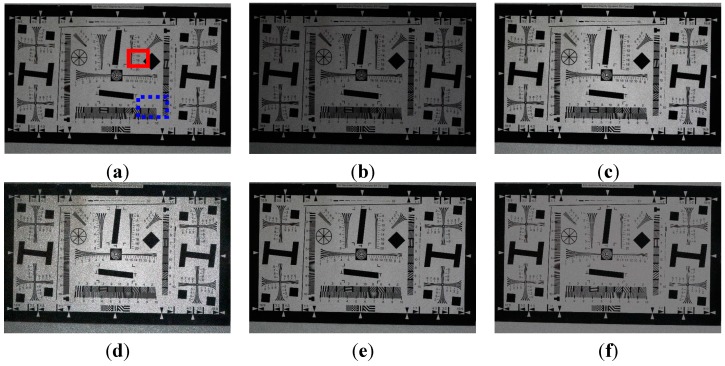
Enhancement results using the ISO 12233 resolution chart image: (**a**) The reference image with ISO 3200 (EV = 0); (**b**) one-step lower exposure image using ISO 1600 (EV = −1); (**c**) enhanced image of (b) using the ordinary two-pixel digital pixel binning; (**d**) enhanced image of (b) using Kim’s algorithm; (**e**) enhanced image of (b) using Jiang’s algorithm; and (**f**) enhanced image of (b) using the adaptive four-pixel digital pixel binning.

Figure 6 shows the experimental results of enhancing the ISO 12233 resolution chart image under 15 lux illumination. Three test images are acquired using different ISO values including 3200 and 1600, fixed aperture size F11, and shutter speed 1/16 s.

**Figure 7:**
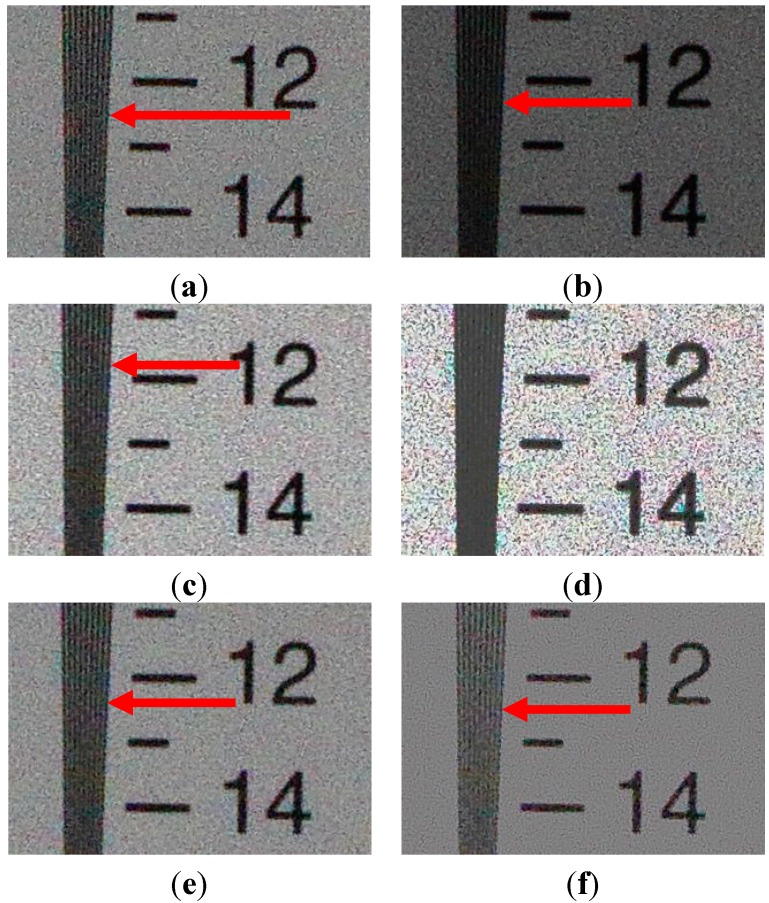
The magnified version of the red box in Figure 6a: (**a**) The reference image with ISO 3200 (EV = 0); (**b**) one-step lower exposure image using ISO 1600 (EV = −1); (**c**) enhanced image of (**b**) using ordinary two-pixel digital pixel binning; (**d**) enhanced image of (**b**) using Kim’s algorithm; (**e**) enhanced image of (**b**) using Jiang’s algorithm; and (**f**) enhanced image of (**c**) using adaptive eight-pixel digital pixel binning.

Figure 6a,b respectively show the normal and one-step lower exposure images. Figure 6c shows the enhanced image of Figure 6b using the ordinary digital pixel binning with 1 × 2 horizontal kernel, such as $\left[\begin{array}{cc} 1 & 1 \end{array}\right]$. Figure 6d,e show enhanced images of Figure 6b using Kim’s and Jiang’s algorithms, respectively. Figure 6f shows the enhanced image of Figure 6b using the proposed algorithm with a maximum binning ratio of 4. For clearer visual comparison, the magnified region of the red box in Figure 6 is shown in Figure 7.

The red arrows in Figure 7 indicate the position where lines can be just resolved with the differential intensity value over 20. Figure 7d is so severely distorted that it is not possible to find a position that has difference of 20 or larger. As shown in Figure 7d–f, the proposed binning algorithm can better preserve vertical edges and suppress noise than existing ordinary image enhancement algorithms. 

For evaluating the performance of noise suppression, Table 1 summarizes the entropy [10] and variance values of a 400 × 400 flat region in the blue dotted box in Figure 6a. The mean value represents the average luminance of the image, and variance the average value of squared difference between the pixel intensity and the corresponding local mean. As shown in Table 1, the higher the ISO value, the more noise in the acquired image. As shown in Table 1 both ordinary binning and Kim’s algorithms (c and d) significantly amplify the noise. On the other hand, the proposed algorithm (f) gives lower entropy and variance values. Although Jiang’s algorithm has a noise filtering function, the noise suppression performance is lower than the proposed algorithm. Noise-adaptive uniform pixel binning can also better suppress noise than ordinary digital pixel binning.

**Table 1:** Mean, entropy, and variance values of six images in Figure 6.

	Mean	Entropy	Variance
(a)	87.4027	5.658747247	49.18756931
(b)	58.8574	5.404929441	40.20226864
(c)	101.7330	6.214999006	69.25368623
(d)	113.9225	6.995119196	117.132437
(e)	87.57096	5.574787129	46.6319541
(f)	91.3899	4.926924468	24.62198825

**Figure 8:**
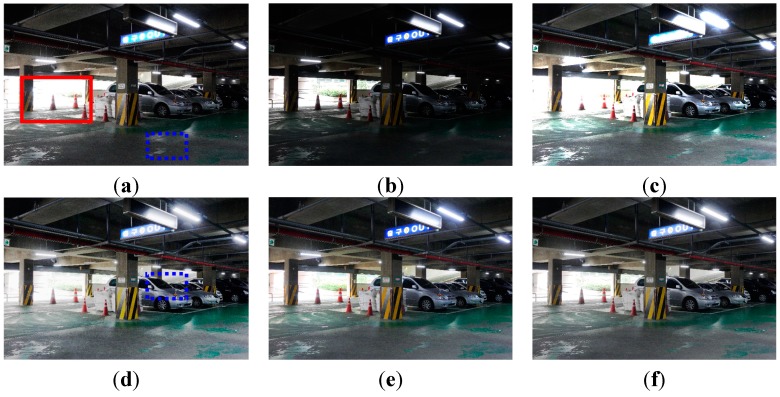
Experimental results using the parking garage image: (**a**) The reference image with ISO 3200 (EV = +1); (**b**) one-step lower image using ISO 800 (EV = −1); (**c**) enhanced image of (b) using ordinary four-pixel digital pixel binning; (**d**) enhanced image of (b) using Kim’s algorithm; (**e**) enhanced image of (b) using Jiang’s algorithm; and (**f**) enhanced image of (b) using the adaptive four-pixel digital pixel binning algorithm.

Figure 8 shows the experimental results of enhancing a typical low-light image acquired under low illumination of under 8 lux. Two input test images are acquired using different ISO values of 3200 and 800, and fixed aperture size F5.6 and shutter speed 1/15 s.

**Figure 9:**
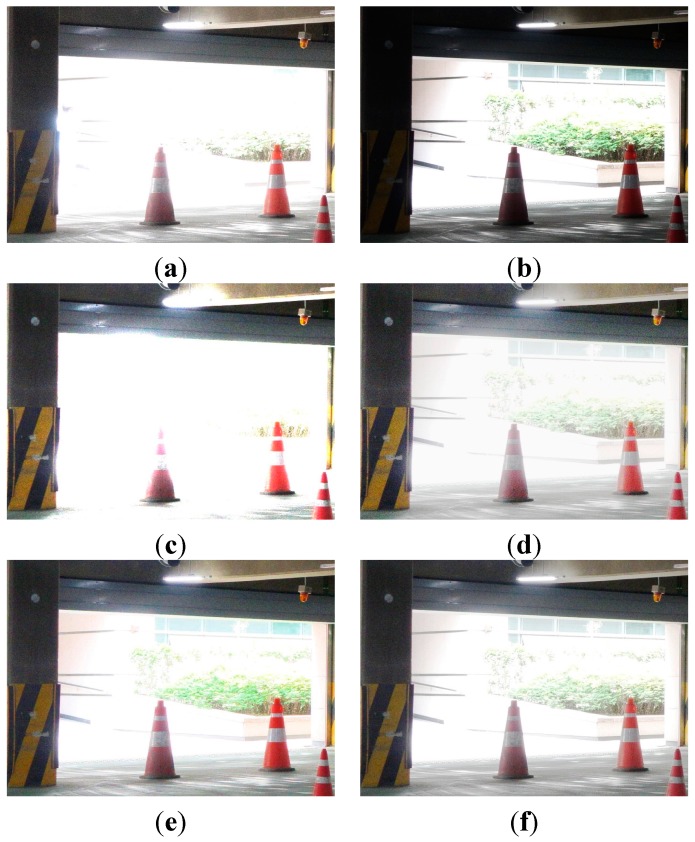
The magnified version of the red box shown in Figure 8a: (**a**) The reference image with ISO 3200 (EV = +1); (**b**) one-step lower exposure image using ISO 800 (EV = −1); (**c**) enhanced image of (b) using ordinary 2 × 2 digital pixel binning; (**d**) enhanced image of (b) using Kim’s algorithm; (**e**) enhanced image of (b) using Jiang’s algorithm; and (**f**) enhanced image of (b) using the adaptive 2 × 2 digital pixel binning algorithm.

Figure 8a,b shows one-step upper and one-step lower exposure images. Figure 8c shows the brightness-enhanced image of Figure 8b using ordinary digital pixel binning with the 2 × 2 binning kernel. Figure 8c,d show brightness-enhanced images of Figure 8b using Kim’s and Jiang’s algorithms, respectively. Figure 8f shows the brightness-enhanced image of Figure 8b using the proposed algorithm with the maximum binning ratio of 4. For clearer visual comparison, the magnified version of Figure 8 is shown in Figure 9.

As shown in Figure 9, the proposed binning algorithm can better preserve details in the saturated region than ordinary algorithms. 

**Table 2:** Mean, entropy, and variance values of four images in Figure 8.

	Mean	Entropy	Variance
(a)	80.8791	5.643978317	49.27466869
(b)	51.1724	3.96713916	6.93515173
(c)	107.4580	5.666662266	51.28301441
(d)	97.76097	6.146823904	66.20613885
(e)	86.25345	5.377880622	40.39314018
(f)	88.7303	5.251893518	37.16049859

To evaluate the performance of noise suppression, Table 2 summarizes the entropy and variance values of a 400 × 400 flat region in the blue dotted box in Figure 8a. As shown by the experimental results in Table 2, the higher the ISO value, the more noise in the acquired image. As shown in Table 2, existing enhancement algorithms (c–e) cannot avoid noise amplification. On the other hand, the proposed algorithm (f) produces lower entropy and variance values.

**Figure 10:**
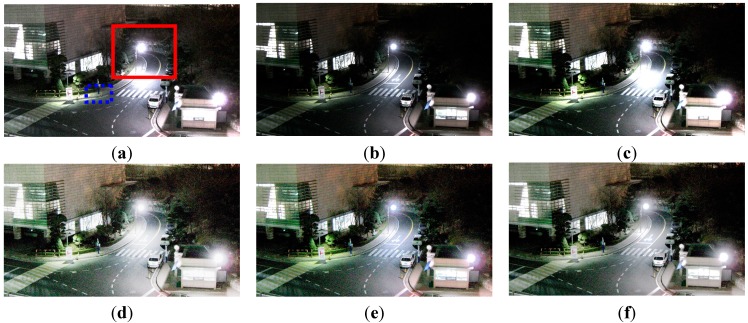
Experimental results using the outdoor image: (**a**) The reference image with 18 dB gain; (**b**) underexposed image with 12 dB gain; (**c**) enhanced image of (b) using ordinary two-pixel digital pixel binning; (**d**) enhanced image of (b) using Kim’s algorithm; (**e**) enhanced image of (b) using Jiang’s algorithm; and (**f**) enhanced image of (b) using the adaptive four-pixel digital pixel binning algorithm.

Figure 10 shows the experimental results of enhancing a typical low-light image acquired by a survaillence camera under low illumination of 3 lux. Two input test images are acquired using different analog front end (AFE) gain of 12 dB and 18 dB, fixed aperture size F1.4, and shutter speed 1/30 s.

**Figure 11:**
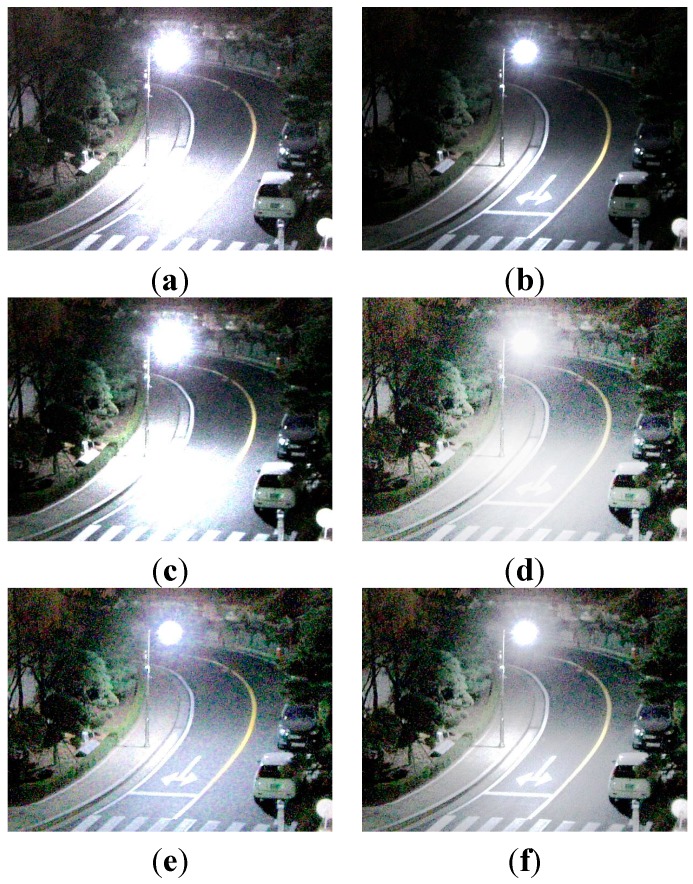
The magnified version of the red box shown in Figure 10a: (**a**) The reference image with 18 dB gain; (**b**) underexposed image with 12 dB gain; (**c**) enhanced image of (b) using ordinary two-pixel digital pixel binning, (**d**) enhanced image of (b) using Kim’s algorithm; (**e**) enhanced image of (b) using Jiang’s algorithm; and (**f**) enhanced image of (b) using the adaptive four-pixel digital pixel binning algorithm.

Figure 10a,b respectively show 18 dB and 12 dB AFE gain images. Figure 10c shows the brightness-enhanced image of Figure 10b using the ordinary digital pixel binning with the 1 × 2 horizontal binning kernel. Figure 10c,d show brightness enhanced images of Figure 10b using Kim’s and Jiang’s algorithms, respectively. Figure 10f shows the brightness-enhanced image of Figure 10b using the proposed algorithm with a maximum binning ratio of 4. For clearer visual comparison, the magnified versions of Figure 10 are shown in Figure 11.

As shown in Figure 10, the proposed binning algorithm can better preserve details in the saturated region than the ordinary algorithms. 

To evaluate noise suppression performance, Table 3 summarizes the entropy and variance values of a 100 × 100 flat region in the blue dotted box in Figure 10a. As shown in Table 3, the higher the AFE gain, the more noise in the acquired image. As shown in Table 3, existing enhancement algorithms (c–e) cannot avoid noise amplification. On the other hand, the proposed algorithm (f) gives lower entropy and variance values.

**Table 3:** Mean, entropy, and variance values of four images in Figure 10.

	Mean	Entropy	Variance
(a)	85.99876	6.275580147	72.95065843
(b)	44.42691	5.547833288	47.37427703
(c)	76.56178	6.389223771	78.242229651
(d)	112.9766	6.012225161	74.25892886
(e)	89.08364	6.083478838	71.54243048
(f)	88.74255	5.839596986	62.86275855

## 5. Conclusions

This paper proposed an adaptive digital pixel binning algorithm in terms of brightness, context, noise, and anti-saturation. In order to solve the noise amplification problem encountered with ordinary brightness amplification methods, the proposed algorithm performs digital pixel binning in an adaptive manner in various aspects. As shown in the experiment, noise-adaptive uniform pixel binning can better suppress noise than the ordinary enhancement methods. Moreover, incorporation of context-adaptive pixel binning can successfully preserve the resolution of high-frequency details in an image. 

The proposed algorithm consists of simple, fundamental arithmetic operations and sorting. The computational complexity depends on the sorting algorithm, which dominates the computational complexity of the entire algorithm. When using the general sorting algorithm, the complexity is O(nlogn), where $n=p^{2}$ represents the number of pixels in the window. Since the proposed algorithm neither requires a frame-memory nor an iterative computation, it is suitable to be embedded in a general image signal processor. Since the proposed algorithm does not affect existing noise reduction or contrast enhancement algorithms, it is very efficient to implement the algorithm along with ISP functions. Future work will combine existing noise reduction algorithms by sharing the hardware to reduce computational complexity.
